# Factors Predicting Abnormal Liver Function Tests Induced by Graves’ Disease Alone: A Retrospective Cohort Study

**DOI:** 10.1097/MD.0000000000000839

**Published:** 2015-05-21

**Authors:** Ruiguo Zhang, Xun Tian, Lan Qin, Xiaoer Wei, Junqi Wang, Jie Shen

**Affiliations:** From the Department of Nuclear Medicine, Tianjin First Central Hospital (RGZ, LQ, JQW, JS); Department of Pathology, Research Institute of Liver Diseases, Tianjin Second People's Hospital, Tianjin (XT); and Department of Radiology, The Sixth Affiliated People's Hospital, Shanghai Jiao Tong University, School of Medicine, Shanghai, China (XEW).

## Abstract

Abnormal liver function tests (LFTs) are often observed in patients with Graves’ disease (GD). To date, there are limited data demonstrating the factors or biochemical indexes contributing to LFT abnormalities in this patient population. The aim of this study was to explore factors predicting abnormal LFTs induced by GD alone. This was a retrospective study of 289 consecutive cases of newly diagnosed and untreated patients with GD. All patients were divided into abnormal LFTs (group A) and normal LFTs (group B). In total, 205 (70.9%) cases were found to have at least 1 LFT abnormality. Among them, the frequencies of ALT, AST, ALP, γ-GTP, TBIL and DBIL abnormalities were 52.7%, 32.2%, 45.9%, 38.5%, 23.4%, 2.9%, respectively, and the number of patients with 1 to 6 hepatic variable abnormalities were 89, 64, 30, 16, 6 and 0, respectively. Logistic regression analysis was used to determine predictive factors contributing to abnormal LFTs. A receiver operating characteristic (ROC) curve was also plotted to verify the accuracy of predictors. In the univariate analysis, patients in group A had significantly higher FT3 concentration (37.5 vs 33.4 pmol/L, *P* = 0.009), FT4 concentration (85.7 vs 77.4 pmol/L, *P* = 0.002) and TRAb level (22.2 vs 17.4 IU/L, *P* < 0.001) when compared with those in group B. Binary logistic regression analysis identified higher FT4 concentration (odds ratio [OR]: 1.017, 95% confidence interval [CI]: 1.005–1.030, *P* = 0.006) and higher TRAb value (OR: 1.038, 95% CI:1.013–1.064, *P* = 0.003) to be independent risk factors predicting abnormal LFTs. The optimal cutoffs for FT4 and TRAb to predict abnormal LFTs were 75 pmol/L and 15 IU/L, respectively, based on ROC analysis.

## INTRODUCTION

Hyperthyroidism is a subcategory of thyrotoxicosis, occurs in approximately 2% of women and 0.2% of men worldwide, and can affect multiple organ systems including the cardiovascular, gastrointestinal and hepatic systems.^1^ Graves’ disease (GD) is the most common cause of hyperthyroidism, and accounts for as many as 50–80% of cases of hyperthyroidism in different regions of the world.^2^ Clinically, it is not uncommon to observe liver function test (LFT) abnormalities in patients with untreated hyperthyroidism. However, the reported prevalence varies between different studies, ranging from 37% to 78%.^3–8^ There are several aspects contributing to hepatic dysfunction in the setting of hyperthyroidism, among them, a wide spectrum has been attributed to hyperthyroidism alone.^9–11^ Mechanistically, some researchers have suggested that excess triiodothyronine (T3) causes hepatic dysfunction by inducing apoptosis via activation of a mitochondrial-dependent pathway, and that this hyperthyroidism-induced apoptosis activates death receptor-mediated pathways.^12,13^ Recently, a study by He et al^3^ demonstrated that elevated TRAb levels may contribute to hepatic dysfunction in patients with GD. Despite these studies, the association between thyroid function indexes and liver injury remains controversial,^4,7,8,14,15^ and to date there are limited data demonstrating the factors or biochemical parameters contributing to LFT abnormalities induced by GD alone.

In the present work, we retrospectively reviewed the clinical data and biochemical indexes in patients with newly diagnosed and untreated GD. We compared patients with abnormal LFTs against those with normal LFTs, and explored the predictors contributing to LFT abnormalities in this patient population.

## MATERIALS AND METHODS

### Subjects

From June 2009 to January 2014, we retrospectively collected data on 289 patients presenting consecutively to the Thyroid Clinic in our hospital who were newly diagnosed with GD. GD was diagnosed on the basis of diffuse goiter, elevated 3- or 24-hour radioiodine uptake (RAIU) of the thyroid, thyrotoxicosis, and/or positive thyrotropin receptor antibodies (TRAb). Patients who were positive for hepatitis B surface antigen and hepatitis C virus antibodies, previously received antithyroid drugs, had evidence of cardiovascular complications or diagnosis of autoimmune liver diseases were excluded. Patients with drug-induced liver dysfunction, nonalcoholic fatty liver disease, excess alcohol consumption were also excluded. This study was approved by the ethical committees of Tianjin First Central Hospital in Tianjin and written informed consent was obtained from each patient.

### Data Collection and Grouping

Data on gender, age, body weight (BW), body mass index (BMI), duration of symptoms, thyroid weight and 24 h-RAIU were collected for all patients. Liver function tests (including serum aspartate aminotransferase [AST], alanine aminotransferase [ALT], alkaline phosphatase [ALP], gammaglutamyl transpeptidase [γ-GTP], total bilirubin [TBIL] and direct bilirubin [DBIL]), thyroid function indexes (including serum free triiodothyronine [FT3], free thyroxine [FT4]) and thyroid autoantibodies (including thyroid peroxidase antibody [anti-TPO], thyroglobulin antibody [anti-Tg] and thyrotropin receptor antibody [TRAb]) were also recorded.

All the patients were divided into an abnormal LFTs group (group A) and a normal LFTs group (group B) depending on whether having at least 1 LFT abnormality.

### Biochemical Analyses

Serum levels of ALT (normal range: 5–35 IU/L), AST (normal range: 5–32 IU/L), ALP (normal range: 40–150 IU/L), γ-GTP (normal range: 5–36 IU/L), TBIL (normal range: 0–17.1 μmol/L), DBIL (normal range: 0–6.8 μmol/L) were measured using a routine automated analyzer (Modular DPP, Roche Diagnostics GmbH, Mannheim, Germany). Serum FT3 (normal range: 3.1–6.8 pmol/L) and FT4 (normal range: 12–22 pmol/L) concentrations, TRAb levels (normal range: 0–1.75 IU/L), anti-TPO (normal range: 0–34 IU/L) and anti-Tg (normal range: 0–115 IU/L) values were measured by chemiluminescent immunoassays (Cobas 6000, Roche Diagnostics GmbH, Mannheim, Germany).

The BMI was calculated as [mass (kg)]/[height (m)]^2^. The 24 h-RAIU value was obtained 24 h after an oral tracer dose (approximately 74 kBq) of 131-radioiodine was administered using a thyroid function instrument (HH-6008, Beijing Hehai Advanced Technology, China). A thyroid scan was performed using 99mTc-pertechnetate via a dual-detector variable-angle gamma camera coupled with a 2-slice CT scanner (Symbia T2, Siemens, Munich, Germany), and thyroid weight was obtained automatically when the region of interest, long and short axis of the thyroid gland were outlined.

### Statistical Analysis

Statistical analysis was performed using SPSS (Statistical Package for Social Sciences) 12.0 for windows (SPSS, Chicago, IL). Continuous variables were expressed as mean ± standard deviation. A chi square test was used to test the gender variable. A Mann-Whitney *U*-test was used to determine differences between all the other continuous variables due to a non-normal distribution. Binary logistic regression analysis with a variable entrance criterion of 0.05 or less was used to determine the factors predicting abnormal LFTs. Receiver operating characteristic (ROC) curves were plotted to verify the accuracy for the prediction of abnormal LFTs. The area under curve (AUC) was used as an estimation of diagnostic accuracy. Missing values for anti-TPO (anti-Tg) and TRAb were assigned to the mean level. All *P* values presented were 2-tailed, and values <0.05 were considered to be statistically significant.

## RESULTS

### Distribution of Liver Variable Abnormalities in Group A

**FIGURE 1:**
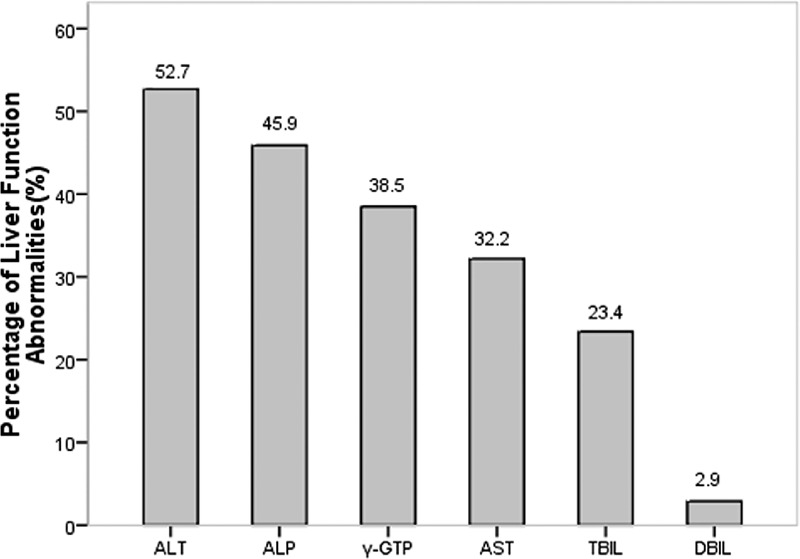
The relative distribution of different LFT abnormalities in GD patients with abnormal LFTs. LFT = liver function test.

Two hundred and five (70.9%) newly diagnosed patients with GD were found to have at least 1 LFT abnormality. Among them, we summarized the percentage of each kind LFT abnormality and the numbers of patients with 1 or more liver variable abnormalities. The frequencies of ALT, AST, ALP, γ-GTP, TBIL and DBIL abnormalities were 52.7%, 32.2%, 45.9%, 38.5%, 23.4%, 2.9%, respectively (Figure 1). The number of patients with 1 to 6 liver variable abnormalities were 89, 64, 30, 16, 6 and 0, respectively.

### Comparison of Patient Characteristics Between Groups

**FIGURE 2:**
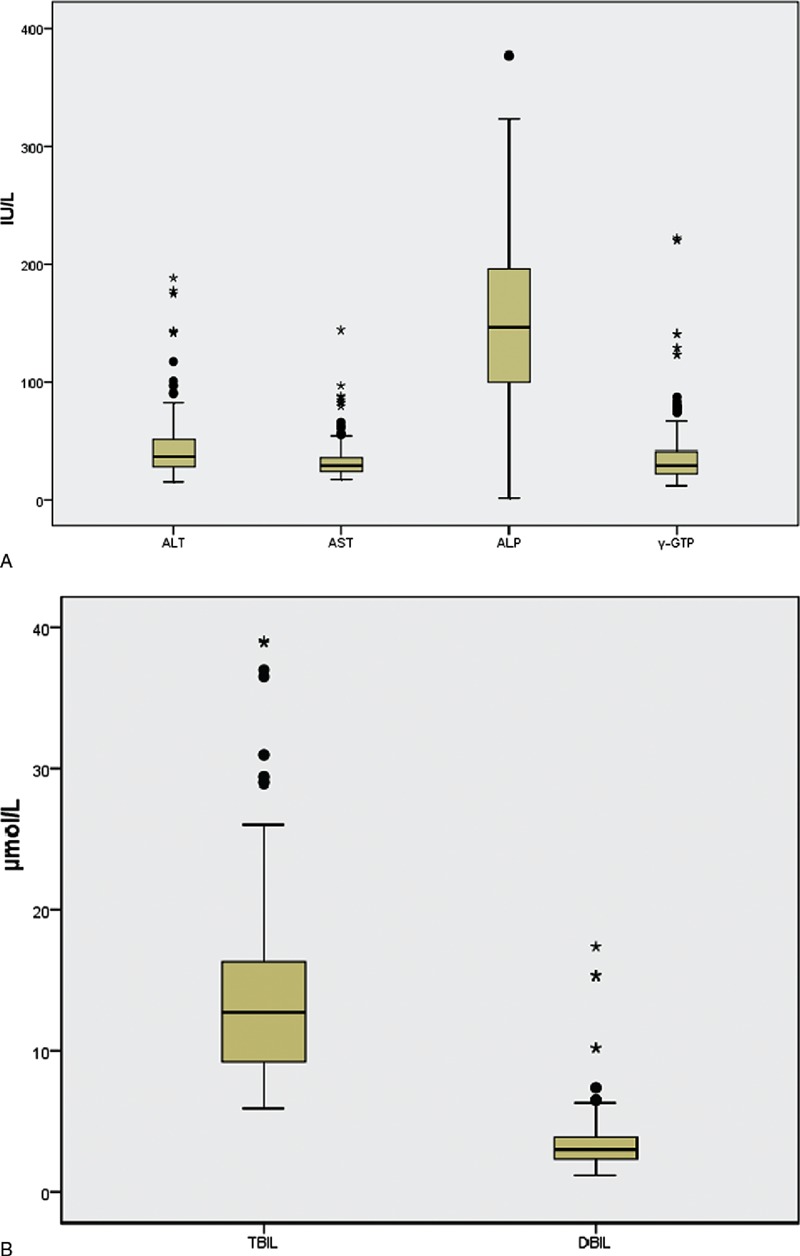
Hepatic enzyme (A) and bilirubin (B) levels in GD patients with abnormal LFTs. LFT = liver function test.

Demographic, clinical and biochemical characteristics of the 289 patients studied are displayed in Table 1. The baseline patient characteristics, including gender, age, BW, BMI, thyroid weight, duration of symptoms, 24 h-RAIU, and thyroid laboratory tests were compared between groups A and B. The results showed that the gender composition was similar (*P* = 0.232) and there were no significant differences found in age, BW, BMI, duration of symptoms, thyroid weight and 24 h-RAIU between the 2 groups (all *P* > 0.05). In terms of thyroid laboratory tests, there was also no significant difference in anti-TPO and anti-Tg values (*P* = 0.599 and *P* = 0.751, respectively). However, subjects at the time of GD diagnosis in group A had significantly higher FT3 concentration (37.5 vs 33.4 pmol/L, *P* = 0.009), FT4 concentration (85.7 vs 77.4 pmol/L, *P* = 0.002) and TRAb values (22.2 vs 17.4 IU/L, *P* < 0.001) when compared with those in group B. Additionally, most of the hepatic enzyme and bilirubin levels were mildly elevated in GD patients with abnormal LFTs (Figure 2).

**TABLE 1:**
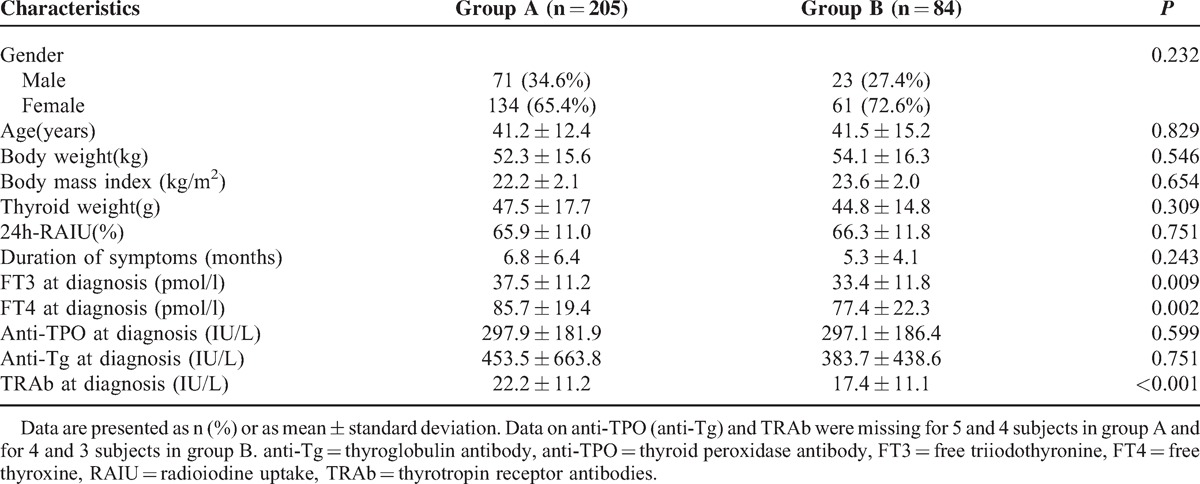
Comparison of Demographic, Clinical and Biochemical Characteristics of Patients Between Groups A and B

### ROC Curves of Predictors of LFT Abnormalities

Table 2 shows a binary logistic regression analysis of predictive factors of abnormal LFTs induced by GD. Variables that were significant in the univariate analysis were entered into the stepwise multivariate logistic regression analysis. The results revealed that patients presenting with higher serum FT4 concentration and TRAb values were more likely to have abnormal LFTs (OR: 1.017 [1.005-1.030] per 1 pmol/l increment, *P* = 0.006; OR: 1.038 [1.013–1.064] per 1 IU/L increment, *P* = 0.003, respectively) but the presenting FT3 concentration at initial diagnosis of GD did not significantly lead to abnormal LFTs, a finding in the univariate model that was not upheld in the multivariate model. Thus, higher serum FT4 concentration and higher TRAb values were independent predictors of LFT abnormalities.

**TABLE 2:**
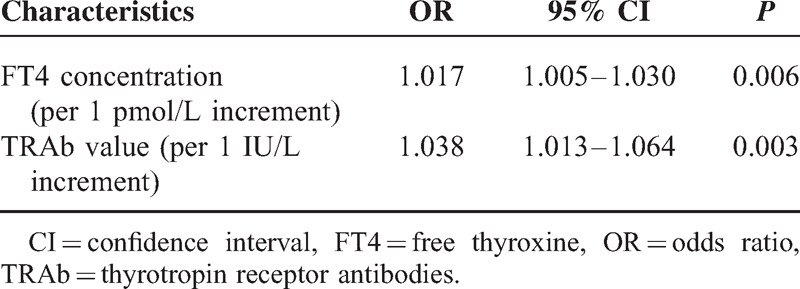
Predictors of Liver Function Test Abnormalities Induced by GD

### ROC Curves of Predictors of LFT Abnormalities

**FIGURE 3:**
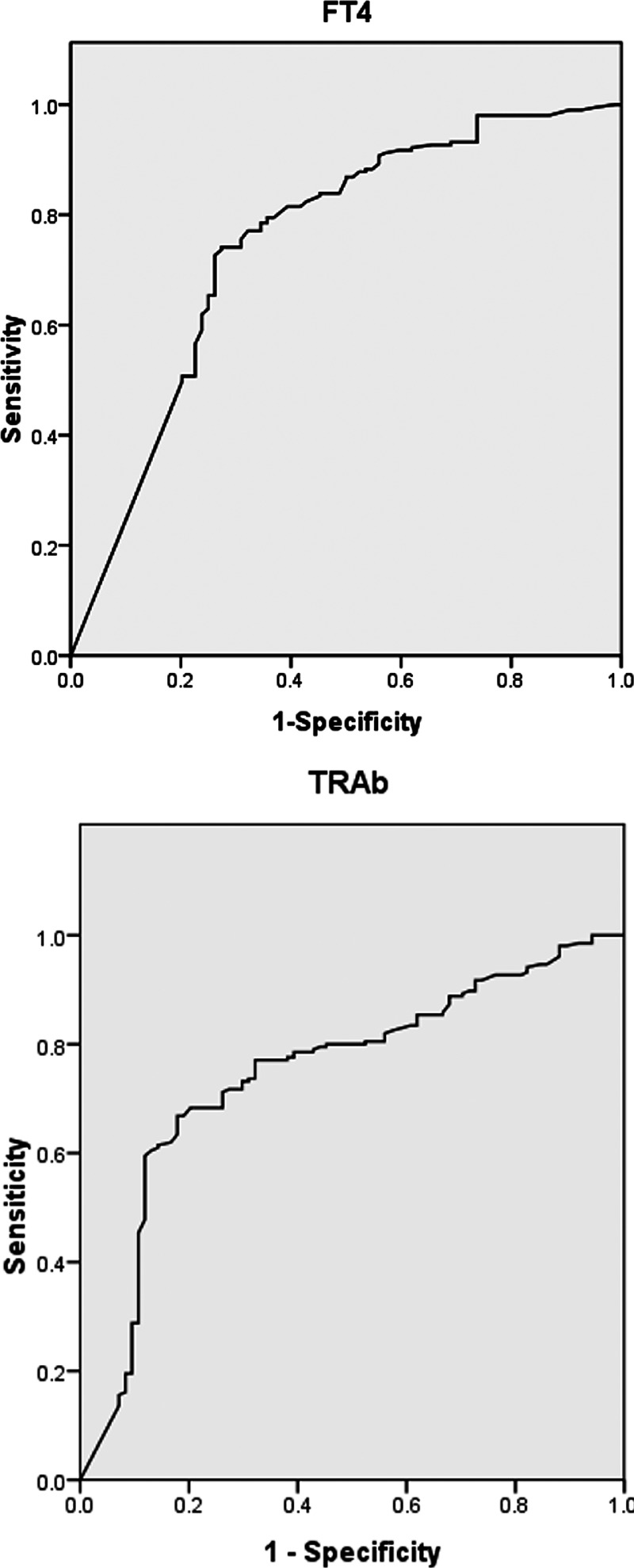
ROC curves for FT4 and TRAb in predicting LFT abnormalities in patients with GD. ROC = Receiver operating characteristic; FT4 = free thyroxine, TRAb = thyrotropin receptor antibodies, LFT = liver function test.

ROC curves were drawn to evaluate the accuracy of FT4 and TRAb in predicting LFT abnormalities induced by GD (Figure 3). The optimal cutoffs were the values yielding maximum sums of sensitivity and specificity from the ROC curves.^16,17^ The results demonstrated that the optimal cutoff value for FT4 was 75.0 pmol/L, at which the sensitivity and specificity were 78.5% and 65.5%, respectively (AUC: 0.750; 95% CI: 0.683–0.816, *P* < 0.001). The optimal cutoff value for TRAb was 15.0 IU/L, at which the sensitivity and specificity were 77.1% and 67.9%, respectively (AUC: 0.748; 95% CI: 0.684–0.811, *P* < 0.001).

## DISCUSSION

Hepatic dysfunction in a patient with hyperthyroidism was first described by Habershon in 1874.^18^ Since then, liver damage or liver malfunction in hyperthyroid patients has been extensively reported.^19–24^ The present study confirmed that abnormal LFTs were frequently observed in patients with newly diagnosed and untreated GD, and most of the liver variable abnormalities in GD patients were mild. Compared with previous reports,^6–8^ this study demonstrated a higher prevalence of abnormal LFTs, with an incidence of 70.9%. This difference in prevalence could be attributed to selection bias of patients (e.g. patients with hyperthyroidism,^7,8^ and untreated or recurrent GD^6^). Our study only recruited the patients with newly diagnosed GD, consistent with other studies reporting a high incidence of liver test abnormalities.^3,5^ Additionally, we speculate that different countries and ethnicities may contribute to differences in the prevalence of LFT abnormalities.

Several aspects contribute to hepatic dysfunction, including liver abnormalities due to hyperthyroidism alone, liver damage related to hyperthyroidism with associated complications (e.g. heart failure), and concomitant liver disease in the setting of hyperthyroidism.^9–11^ Certainly, anti-hyperthyroidism treatments, especially antithyroid drug therapy, may themselves cause hepatic dysfunction.^22,25–27^ A wide spectrum of liver abnormalities have been attributed to hyperthyroidism alone. Mechanistically, in a rat model, excess T3 causes hepatic dysfunction by inducing apoptosis via activation of a mitochondrial-dependent pathway,^13^ and this hyperthyroidism-induced apoptosis involved the activation of death receptor-mediated pathways, including p75.^12^ Recently, it was demonstrated that elevation of TRAb also contributed to hepatic dysfunction in patients with GD.^3^ However, to date, the exact pathogenesis of liver damage in pure hyperthyroidism has not been fully elucidated.

The high frequency of liver damage in patients with untreated GD highlights the importance of determining predictive factors of LFT abnormalities in this patient population. In the present study, we did not find any differences in gender, age and duration of symptoms between the 2 groups. Moreover, we also found that values of thyroid weight, BW, BMI, 24 h-RAIU, anti-TPO and anti-Tg were similar when comparing patients with abnormal LFTs against those with normal LFTs. Consistent with previous findings,^7^ in our study the free-T3 and free-T4 levels were observed to be more elevated in patients with abnormal LFTs, which may be due to excess thyroid hormone causing hepatic tissue hypoxia via increased hepatic and splanchnic oxygen requirement. However, other studies have suggested that liver enzyme levels do not correlate with those of thyroid hormones,^4,8,19^ and even low FT4 concentrations were associated with hepatic steatosis.^14,15^ We speculate that selection bias of recruited patients is the main reason for these differences. Their study populations were patients with hyperthyroidism (including those with hyperactive nodules),^4,8^ or inpatients,^15,19^ and hepatic steatosis was defined using serum transaminase concentrations^15^ or both sonographic and serum ALT criteria.^14^ Contrastingly, our study only recruited newly diagnosed and untreated GD patients. Additionally, the different thyroid function indexes investigated [serum T3 and T4^4,8^], to some extent, could lead to the inconsistent results in the literature. Moreover, our study also revealed that patients with abnormal LFTs had higher TRAb levels, as recently reported.^3^

The present study used a multivariate logistic analysis, revealing that GD patients with high levels of FT4 and/or TRAb had a greater risk of developing LFT abnormalities. The sensitivity and specificity of serum FT4 using 75.0 pmol/L as cut-off for the prediction of LFT abnormalities were 78.5% and 65.5%, and the sensitivity and specificity of serum TRAb using 15.0 IU/L as cut-off were 77.1% and 67.9%, respectively. Thyroid-stimulating hormone (TSH) mediates its function through highly specific interactions with the thyrotropin receptor (TSHR).^28^ Interestingly, TSHR is a target of autoimmune antibodies, leading to dysfunction of the thyroid gland in many autoimmune thyroid disorders, such as GD.^29,30^ TSHR mRNA expression in human liver tissues was first described via PCR in 2002,^31^ and Zhang et al^32^ found that TSHR was not only present and functional in hepatocytes, but hepatic TSHR mRNA had the same sequence as that of thyroid-derived mRNA. We speculate that TSH, besides its classical role in regulating the thyroid function, also acts on the TSHR in hepatocytes, which may lead to abnormal LFTs. Further studies are required to elucidate the direct effects and the mechanism of TSHR in hepatocytes on liver tissue and hepatic function.

This retrospective analysis had some inherent limitations associated with this type of study. Our aim was to explore the predictors of LFT abnormalities induced by GD. Although we adopted rigorous inclusion and exclusion criteria and none of the patients in our study had diagnosis of autoimmune liver diseases, testing was not universally conducted to specifically exclude this possibility. Additionally, we only selected FT3 and FT4 as the thyroid function indexes and did not include T3 and T4, while excess T3 has been shown to affect hepatic function.^13^ Therefore, larger prospective studies will need to be performed to confirm these preliminary results.

In conclusion, the present study of a relatively large consecutive cohort indicates that abnormal LFTs in patients with newly diagnosed and untreated GD are common and mild. Higher serum FT4 concentration and TRAb values are independent risk predictors of LFT abnormalities. The optimal cutoffs for FT4 and TRAb to predict LFT abnormalities were 75 pmol/L and 15 IU/L, respectively.
